# MAGED2 Enhances Expression and Function of NCC at the Cell Surface via cAMP Signaling Under Hypoxia

**DOI:** 10.3390/cells14030175

**Published:** 2025-01-23

**Authors:** Aline Radi, Sadiq Nasrah, Michelle Auer, Aparna Renigunta, Stefanie Weber, Elie Seaayfan, Martin Kömhoff

**Affiliations:** Department of Pediatrics, University Hospital Giessen and Marburg, Philipps University Marburg, 35043 Marburg, Germany; radi@staff.uni-marburg.de (A.R.); sadiq.nasrah@med.uni-muenchen.de (S.N.);

**Keywords:** *MAGED2*, NCC, Bartter syndrome, protein trafficking, endocytosis, exocytosis, lysosomal degradation, cAMP/PKA pathway

## Abstract

Mutations in *MAGED2* cause transient antenatal Bartter syndrome (tBS) characterized by excessive amounts of amniotic fluid due to impaired renal salt transport via *NKCC2* and NCC, high perinatal mortality, and pre-term birth. Surprisingly, renal salt handling completely normalizes after birth. Previously, we demonstrated that, under hypoxic conditions, *MAGED2* depletion enhances endocytosis of GalphaS (Gαs), reducing adenylate cyclase (AC) activation and cAMP production. This impaired cAMP signaling likely contributes to the dysfunction of salt transporters *NKCC2* and NCC, explaining salt wasting and the subsequent recovery with renal oxygenation after birth. In this study, we show that *MAGED2* depletion significantly decreases both total cellular and plasma membrane NCC expression and activity. We further demonstrate that *MAGED2* depletion disrupts NCC trafficking by reducing exocytosis, increasing endocytosis, and promoting lysosomal degradation via enhanced ubiquitination. Additionally, forskolin (FSK), which increases cAMP production by activating AC, rescues NCC expression and localization in *MAGED2*-depleted cells. Conversely, *MAGED2* overexpression increases NCC expression and membrane localization, although this effect is diminished in Gαs-depleted cells, indicating that Gαs acts downstream of *MAGED2*. In summary, our findings reveal the essential role of *MAGED2* in regulating NCC function and trafficking under hypoxic conditions, providing new insights into the mechanisms behind salt loss in tBS and identifying potential therapeutic targets.

## 1. Introduction

Bartter syndrome (BS) comprises a group of rare hereditary salt-losing tubulopathies caused by mutations in genes encoding ion transporters and/or regulatory subunits expressed within cells of the thick ascending loop of Henle (TAL), where approximately 20% of filtered sodium is reabsorbed. These mutations already impair renal salt reabsorption in the affected fetus, causing excessive formation of amniotic fluid (termed “polyhydramnios”) and leading to preterm birth. The hallmark biochemical signature of Bartter syndrome involves hypokalemic, hypochloremic metabolic alkalosis with hyperaldosteronism. These symptoms arise from defective salt reabsorption in the TAL, leading to compensatory potassium and proton secretion in response to enhanced distal delivery of sodium, which promotes excretion of potassium. Bartter syndrome is further aggravated by secondary hyperaldosteronism due to defective salt-sensing of the macula densa [1,2,3]. Bartter syndrome V recently emerged as the most severe yet transient variant, caused by mutations in the X-linked gene *MAGED2* [4,5,6]. Clinically, tBS is characterized by early severe polyhydramnios, fetal macrosomia, preterm delivery, postnatal polyuria, and high perinatal mortality. The severity of tBS, as reflected by earlier onset of polyhydramnios and delivery compared to Bartter syndrome 1–4, results from the compromised compensatory reabsorption of sodium and chloride downstream of the TAL in the distal convoluted tubule via NCC. Similar to *NKCC2*, NCC expression in fetal renal sections in patients with tBS is reduced, with a lack of its expression at the plasma membrane [5].

*MAGED2*, a member of the melanoma-associated antigen (MAGE) family, plays a crucial role in stress response pathways [7]. Under cellular stress, including hypoxia, *MAGED2* acts as a master regulator of cellular cyclic adenosine 3′,5′-monophosphate (cAMP) levels by preventing *MDM2*-dependent ubiquitination of Gαs, a stimulatory subunit of G-protein coupled receptors [8]. Regulation of cAMP, a key second messenger in cellular responses to hormones and neurotransmitters, is essential for the proper expression and function of renal salt transporters like *NKCC2* and NCC through the activation of protein kinase A (PKA) [9]. Notably, the spontaneous recovery from renal salt wasting in tBS coincides with a developmental increase in renal oxygenation, suggesting that hypoxia-sensitive *MAGED2*-mediated cAMP/PKA activation is a critical determinant of the disease’s transient nature.

In transient Bartter syndrome, dysregulation of NCC is evidenced by its aberrant expression in the fetal kidney in a tBS patient, a finding corroborated in vitro by showing significantly increased NCC cell surface expression upon *MAGED2* overexpression [5]. Hence, we investigated the impact of *MAGED2* depletion and overexpression on NCC trafficking under hypoxia, which is characteristic of fetal DCT [10]. NCC, a member of the cation-chloride cotransporter superfamily (CCC), is encoded by the solute carrier family 12-member 3 (*SLC12A3*) gene and comprises 12 transmembrane domains. It functions as a glycosylated homodimer expressed solely in the distal convoluted tubule [11]. NCC plays a pivotal role in maintaining electrolyte balance and regulating blood pressure [12]. In Gitelman syndrome, loss of function mutations of *SLC12A3* lead to salt loss and hypokalemia [13], while, on the contrary, its constitutive activation by upstream regulatory kinases or E3 ubiquitin ligases cause hypertension hyperkalemia in Gordon’s syndrome [14,15].

In addition to the critical role of the NCC transporter in tBS, in a pre-eclampsia mouse model induced by Nω-nitro-L-arginine methyl ester hydrochloride (L-NAME), increased renal expression of HIF1α was observed, accompanied by elevated levels of total and phosphorylated NCC, contributing to hypertension [16]. These findings align with our previous observations, where *MAGED2* depletion led to reduced HIF1α accumulation during hypoxia [17]. Furthermore, a recent study reported an increase in urinary exosomal NCC, similar to *NKCC2*, in rats with acute kidney injury (AKI) induced by candesartan combined with a low-salt diet [18]. Of note, *MAGED2* is upregulated in the DCT in a murine AKI model [19]. Taken together, these results indicate that *MAGED2* may regulate NCC under hypoxic conditions.

Based on our recent discovery of *MAGED2*’s essential role in regulating Gαs ubiquitination and PKA activity under stress conditions including hypoxia [8,17], the present study addresses the mechanisms by which *MAGED2* regulates NCC trafficking under hypoxic conditions. Hypoxia, which is prevalent in fetal kidneys until approximately gestational week 30, coincides with the onset of spontaneous recovery observed in tBS. By using biotinylation and protein trafficking assays, we revealed significant reduction in total NCC, as well as membrane-localized NCC, upon *MAGED2* depletion. We identified both decreased NCC exocytosis and enhanced endocytosis in *MAGED2*-depleted cells. Additionally, *MAGED2* depletion enhanced lysosomal degradation of NCC by increasing its ubiquitination. Interestingly, forskolin, a potent activator of adenylate cyclase, mitigated the effects of *MAGED2* depletion, underscoring the significance of the cAMP/PKA pathway in this regulatory paradigm.

In summary, our findings highlight the critical role of *MAGED2* in modulating NCC trafficking and stability under hypoxic conditions, offering deeper insights into the pathophysiological mechanisms underlying tBS. This enhanced understanding not only bridges existing knowledge gaps, but also paves the way for potential therapeutic strategies targeting *MAGED2*-mediated pathways to ameliorate renal salt wasting in affected individuals.

## 2. Materials and Methods

### 2.1. Materials

The key materials and resources are listed in Table 1.

### 2.2. Plasmid Constructs and Site Directed Mutagenesis

The NCC cDNA was subcloned into the mammalian expression vector pTargeT (Promega, Walldorf, Germany) using BamHI and NotI as restriction enzymes. The generation of the 3×HA-tagged NCC was conducted by Site-Directed Mutagenesis, using the pTargeT NCC as a template, following the NEB Q5^®^ Site-Directed Mutagenesis Kit protocol (New England Biolabs, Ipswich, MA, USA). The 3×HA-tagged NCC plasmid was confirmed by sequencing.

### 2.3. Cell Culture, Hypoxia Induction and Transfections

Human Embryonic Kidney (HEK293) and HeLa cells were maintained in DMEM GlutaMax medium, which was supplemented with 10% fetal bovine serum (Invitrogen, Dreieich, Germany), as well as penicillin (100 units/mL) and streptomycin (100 units/mL), at 37 °C in a humidified 5% CO_2_ incubator. Growth conditions such as seeding, cell passage, etc., were uniform in each experiment for both control and experimental samples. Hypoxia was induced chemically in serum-free DMEM containing 300 µM cobalt chloride (CoCl_2_) for 14–16 h or physically in serum-free DMEM within a sealed chamber (Billups-Rothenberg, Inc., San Diego, CA, USA, Cat. SFM-3001) filled with a mixture of 1% O_2_, 5% CO_2_, and 94% N_2_, and maintained at 37 °C in a humidified 5% CO_2_ incubator for 14–16 h [8,21,22]. Experiments were conducted under physical hypoxia, except for the assays, to study endocytosis, exocytosis, and sodium uptake. Hypoxia induction in both methods was validated by detecting HIF1α protein expression via Western blotting. For transfections, reverse transfections of control and specific siRNA were achieved using DharmaFECT 4 as the reagent, and 3×HA-NCC and *MAGED2* expression plasmids were transfected with DreamFect Gold using the manufacturer’s specifications.

### 2.4. Biotinylation

Following hypoxia treatment, confluent HEK293 cells were washed twice with PBS containing 1 mM MgCl_2_ and 0.1 mM CaCl_2_ (PBS^++^). Cells were then incubated at 4 °C for 30 min in PBS^++^ that contained 1 mg/mL EZ-Link™ sulfo-NHS-LC-biotin, followed by three washes with quenching buffer (100 mM glycine in PBS^++^) for 10 min. After three additional PBS^++^ washes, cells were lysed for 45 min at 4 °C in solubilizing buffer (150 mM NaCl, 5 mM EDTA, 3 mM KCl, 120 mM Tris/Hepes, pH 7.4, 1% Triton X-100) containing protease inhibitors (Sigma, Schnelldorf, Germany). The protein level was measured by BCA. An aliquot of the normalized total lysate was taken to assess the protein total expression, while the remaining lysates were incubated with streptavidin-agarose beads overnight at 4 °C. Following this incubation, samples were centrifuged at 13,000 rpm for 5 min to separate the intracellular fraction. Streptavidin-agarose beads were washed seven times with solubilizing buffer. The bound proteins were eluted by heating the pellets at 95 °C for 10 min in solubilizing and denaturing buffer. The samples were then stored at −20 °C. Each fraction was subsequently analyzed by SDS-PAGE and Western blotting using specific primary and fluorescence-conjugated secondary antibodies.

### 2.5. Immunocytochemistry

HeLa cells were grown on Poly-L-lysine-coated chamber slides to promote adherence. After biotinylation, cells were fixed using 4% paraformaldehyde in PBS for 30 min at 4 °C, permeabilized with 0.1% Triton X-100 for 5 min at 4 °C. The non-specific binding site were blocked with DAKO (antibody diluent with background-reducing components) for 30 min at room temperature. Fixed cells were then incubated with the primary antibody (mouse anti-HA, 1:50) in DAKO for 1 h at RT. Biotinylated membrane proteins and mouse anti-HA were visualized with Alexa 488-coupled streptavidin (1:500) and Alexa 555-coupled secondary antibody (1:1000), respectively. Cells were mounted in VECTASHIELD Antifade Mounting Medium containing DAPI (Vector Laboratories, Newark, CA, USA) and examined under a ZEISS Apotome microscope with a 40× objective lens (Carl Zeiss, Oberkochen, Germany).

### 2.6. Exocytosis Assay

The exocytic insertion of NCC in HEK293 cells was assessed by first saturating NHS-reactive sites on the cell surface with sulfo-NHS-acetate for 2 h at RT to block any available amino groups. After surface saturation, cells were returned to incubate at 37 °C to resume cellular trafficking, allowing exocytosis to occur for 30 min. To stop the process of protein trafficking, cells were then incubated at 4 °C. Subsequently, the newly inserted membrane proteins were labeled with sulfo-NHS-LC-biotin for 30 min at 4 °C. The cells were then lysed, and the newly inserted surface proteins were affinity-precipitated using streptavidin-coupled agarose beads. Isolated proteins were analyzed via SDS-PAGE and Western blotting to evaluate exocytic insertion (Figure S1A).

### 2.7. Endocytosis Assay

Cells were treated with 300 µM CoCl_2_ overnight to create hypoxic conditions. The next day, membrane protein trafficking was halted by cooling the cells to 4 °C. Plasma membrane proteins were labeled with cleavable sulfo-NHS-SS-biotin (Thermo Scientific, Dreieich, Germany), which binds to the extracellular amino groups. After biotinylation, cells were then incubated at 37 °C to resume cellular trafficking, allowing endocytosis to occur for 15 or 30 min. To stop the process of protein trafficking, cells were then returned to 4 °C, and treated twice with L-glutathione reducing reagent (50 mM glutathione, 75 mM NaCl, 75 mM NaOH, 10 mM EDTA), each for 15 min, to cleave disulfide bonds, thus removing biotin from the cell surface proteins. This step ensured that only the biotinylated proteins that had been endocytosed remained labeled. The biotinylated, endocytosed proteins were then isolated using streptavidin-agarose beads, which specifically bind to biotin. The bound proteins were then eluted using SDS sample buffer, and analyzed via SDS-PAGE and Western blotting to assess the extent of endocytosis and protein trafficking (Figure S1B).

### 2.8. Lysosomal Inhibition Assays

HEK293 cells were transfected with either control or *MAGED2* siRNA using DharmaFECT transfection reagent and overexpressed with 3×HA-tagged NCC plasmid the next day. Cells were placed in a hypoxia chamber to induce hypoxia overnight with the lysosome inhibitor 100 μM leupeptin. Cells were washed three times in ice-cold PBS, lysed via sonication in RIPA buffer (50 mM Tris-HCl, 150 mM NaCl, 1 mM EDTA, 0.1% SDS, 1% Triton X-100), and processed for protein quantification using BCA to be analyzed with Western blot.

### 2.9. Measurement of NCC-Mediated Sodium Uptake Using CoroNa Green

HEK293 cells were transfected with either control or *MAGED2* siRNA using DharmaFECT. The following day, cells were overexpressed with 3×HA-tagged NCC using DreamFect Gold, with non-transfected cells serving as controls. To measure sodium uptake, cells were treated with different sodium transporter blockers (1 mM ouabain, 200 μM bumetanide, and 100 μM amiloride) with or without 200 μM hydrochlorothiazide (HCTZ, NCC inhibitor) for 30 min at RT. Subsequently, cells were loaded with 10 μM CoroNa Green, a fluorescent intracellular sodium indicator that is permeable to membrane, for 1 h at 37 °C in a humidified 5% CO_2_ incubator. Post-incubation, cells were washed three times with ice-cold PBS to remove any unbound dye, then lysed via sonication in a buffer composed of 50 mM MOPS, 100 mM KCl, and 1% NP-40. The fluorescence was assessed using a Tecan fluorescence plate reader and normalized to protein concentration.

### 2.10. Ubiquitination Assay

HEK293 cells, subjected to *MAGED2* knockdown and NCC overexpression as previously described, were placed in a hypoxia chamber overnight. Following exposure, cells were lysed in RIPA buffer containing 50 mM Tris-HCl, 150 mM NaCl, 1 mM EDTA, 0.1% SDS, 1% Triton X-100, and 100 mM N-ethylmaleimide. The lysates were clarified by centrifugation at 13,000 rpm for 15 min. Protein concentrations were measured and normalized using the Pierce™ BCA Protein Assay Kit (Thermo Scientific, Dreieich, Germany). The normalized samples were then subjected to immunoprecipitation with Dynabeads protein G conjugated to an anti-HA antibody. After a 1 h incubation at RT with magnetic beads conjugated to the specified antibody, the immune complexes were washed three times with PBS (Invitrogen, Dreieich, Germany). The proteins were then denatured in loading buffer, resolved on a 7.5% TGX Stain-Free gel, and detected using the appropriate primary antibodies followed by fluorescence-labeled secondary antibodies, according to the established protocols.

### 2.11. Western Blotting

Cells were rinsed three times with ice-cold PBS and lysed in a lysis buffer (50 mM Tris (pH 7.4), 5 mM EDTA, 150 mM NaCl, 1% Triton X-100, and protease inhibitors). Cell lysates were clarified by centrifugation at 13,000× rpm for 15 min. Protein concentrations were measured using the Pierce™ BCA Protein Assay Kit (Thermo Scientific, Dreieich, Germany). Proteins were resolved on 7.5% TGX Stain-Free gels (Bio-Rad, Dreieich, Germany) and transferred to nitrocellulose membranes via the Trans-Blot Turbo Transfer System (Bio-Rad, Dreieich, Germany). Protein detection was carried out with fluorescently labeled antibodies, and StarBright Blue 520 and 700 (Bio-Rad, Dreieich, Germany). Blots were visualized with the ChemiDoc MP system (Bio-Rad, Dreieich, Germany), and the gray density of the Western blots was quantified using ImageJ software (NIH).

### 2.12. Statistical Analyses

Results are presented as mean ± SEM. Comparisons between the means were conducted using unpaired Student’s *t*-tests, with statistical analyses executed via GraphPad Prism 9 software. Statistical significance is indicated as follows: * *p* ≤ 0.05, ** *p* ≤ 0.01 and *** *p* ≤ 0.001.

## 3. Results

### 3.1. MAGED2 Regulates Total and Membrane Expression of NCC Under Hypoxic Conditions

**Figure 1:**
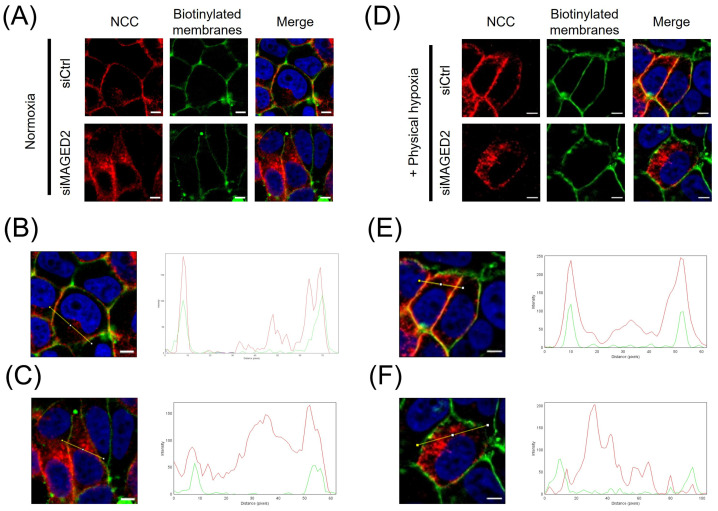
***MAGED2* promotes membrane expression of NCC, particularly under hypoxic conditions.** Immunolocalization of NCC proteins under normoxic (**A**) and hypoxic (**D**) conditions, in the presence or the absence of *MAGED2*. HeLa cells were co-transfected with a 3×HA-NCC construct and either control or *MAGED2* siRNA. Forty-eight hours post-transfection, cells were exposed to normoxia (**A**) or hypoxia (1% O_2_) overnight (**D**). Membrane proteins were biotinylated at 4 °C. Scale bars: 5 μm. The distribution of NCC (red) and biotinylated membrane proteins (green) was analyzed using the “RGB profile plot” plugin in ImageJ. Panels B and C depict normoxia, and E and F represent hypoxia, with comparisons made between the absence (**B**,**E**) and presence (**C**,**F**) of *MAGED2* siRNA.

**Figure 2:**
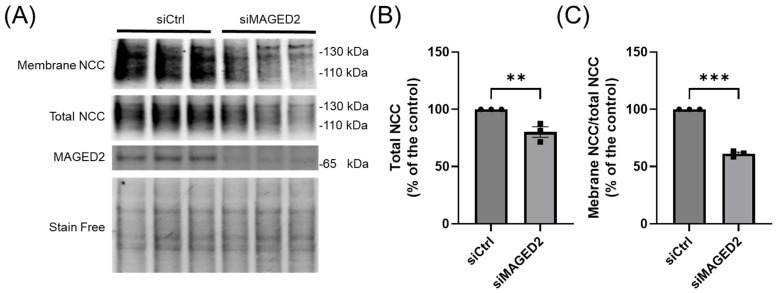
***MAGED2* promotes membrane expression and prevents degradation of NCC under hypoxic conditions.** Co-transfection of HEK293 cells with 3×HA-NCC and either control or *MAGED2* siRNA. At 24–48 h post-transfection, cells were subjected to physical hypoxia (1% O_2_) overnight. Surface proteins were biotinylated and isolated via streptavidin-agarose precipitation. (**A**) Surface NCC was detected by Western blot using an anti-HA antibody. Parallel SDS-PAGE and Western blotting were performed on total cell extracts to assess total NCC and *MAGED2* expression. (**B**) Densitometric analysis of total NCC and (**C**) the ratio of membrane-bound NCC to total NCC is shown. Bar graphs represent mean ± SEM with statistical significance determined by an unpaired one-sided Student’s *t*-test, ** *p* ≤ 0.01, *** *p* ≤ 0.001. The results shown are representative of three independent experiments.

To gain further insight into the role of *MAGED2* in renal salt reabsorption associated with Bartter syndrome type V (tBS), we initially investigated the effect of *MAGED2* on the localization of the NCC under normoxic and hypoxic conditions in HEK293 cells. The cellular distribution of transiently expressed 3xHA-NCC in both control and *MAGED2*-depleted cells was assessed using immunofluorescence. Our findings demonstrated that under normoxic conditions, *MAGED2* knockdown induces a change in the cellular distribution of NCC, specifically leading to a significant reduction in its membrane expression (Figure 1B,C). This observation is consistent with our previously published data [5], which demonstrated that the overexpression of *MAGED2* enhances NCC membrane localization. Under hypoxic conditions, the effect of *MAGED2* knockdown on NCC membrane localization was even more pronounced (Figure 1E), likely due to the increased level of cellular stress compared to the stress induced by transient transfection of transmembrane proteins [23,24]. A similar effect of *MAGED2* depletion under hypoxia on NCC expression was observed in Western blot analysis (Figure 2A,B), which revealed a reduction in total NCC expression and a greater effect on NCC membrane expression, as judged by the reduced ratio of membrane NCC to total NCC. These results suggest that *MAGED2* regulates both the degradation and trafficking pathways of NCC.

### 3.2. MAGED2 Regulates the Function of NCC Under Hypoxic Conditions

**Figure 3:**
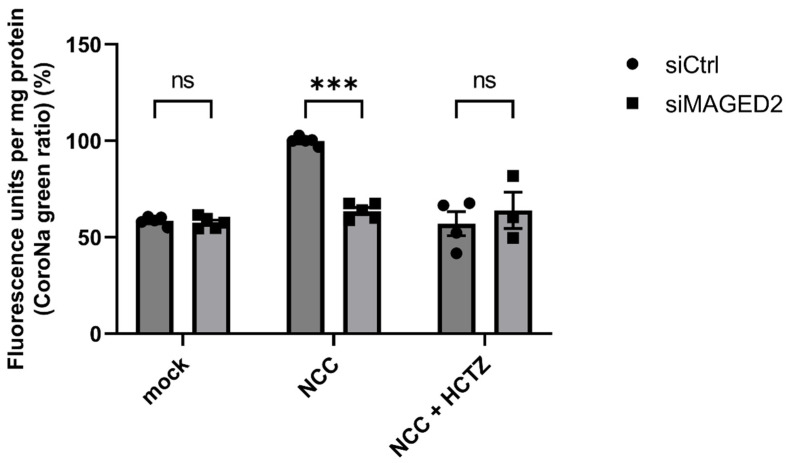
***MAGED2* promotes sodium uptake by NCC in HEK293 cells under hypoxic conditions.** HEK293 cells were transfected with either control or *MAGED2* siRNA. At 90% confluency, cells were transfected with 3×HA-tagged NCC, while non-transfected cells served as mock controls. Cells were exposed to hypoxia mimetic (300 µM CoCl_2_) overnight before sodium uptake assessment. Sodium uptake was assessed following treatment with sodium transporter inhibitors (1 mM ouabain, 200 μM bumetanide, 100 μM amiloride) with or without 200 μM HCTZ for 30 min at room temperature. Cells were subsequently loaded with 10 μM CoroNa Green, a fluorescent sodium indicator, for 1 h at 37 °C in a humidified 5% CO_2_ incubator. After incubation, cells were washed with PBS and lysed by sonication. Fluorescence intensity was measured with a Tecan fluorescence plate reader and normalized to the respective protein concentration. Bar graphs represent mean ± SEM with statistical significance determined by an unpaired two-sided Student’s *t*-test, ns, not significant, *** *p* ≤ 0.001. The results shown are representative of four (NCC + HCTZ) or five (mock and NCC) biological replicates.

We then explored whether the reduction in NCC levels translated into diminished transporter activity. To this end, we conducted functional studies utilizing CoroNa Green AM (CoroNa), a fluorescent intracellular sodium indicator that is permeable to membrane, to evaluate NCC-mediated sodium uptake in control and *MAGED2*-depleted HEK293 cells. Cells were incubated in PBS supplemented with ouabain (Na+-K+-ATPase inhibitor), bumetanide (NKCC blocker), amiloride (Na+/H+ antiporter blocker), and hydrochlorothiazide (HCTZ, NCC blocker). Subsequently, the cells were loaded with CoroNa Green in the presence of these same inhibitors. NCC-transfected cells showed higher fluorescence units per mg protein compared to non-transfected (mock) cells, confirming the high sodium content in the presence of NCC. The inhibition of NCC by HCTZ abolished this increase in intracellular sodium, demonstrating that the elevated fluorescence resulted from NCC activity. *MAGED2*-depleted cells had significantly lower intracellular sodium compared to control cells, indicating decreased NCC activity (Figure 3). These findings suggest that *MAGED2* is crucial for both the proper expression of NCC and also for its transport activity.

### 3.3. Forskolin Reverses Reduced Expression of Total and Membrane Bound NCC Caused by MAGED2 Depletion

**Figure 4:**
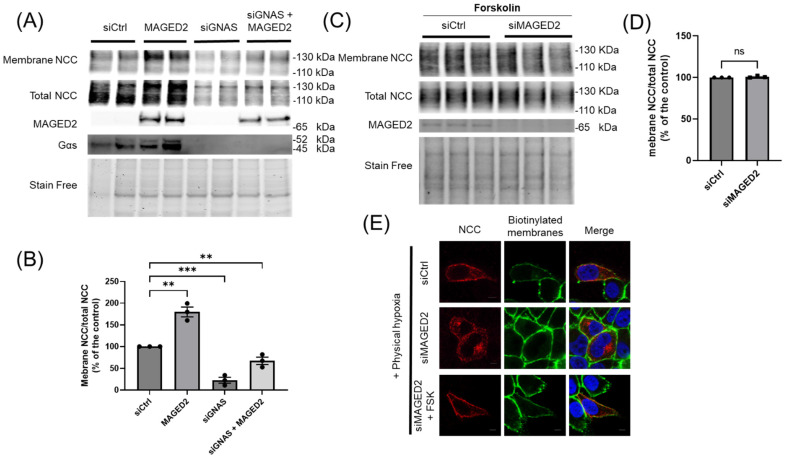
***MAGED2* promotes NCC membrane expression via Gαs signaling in HEK293 cells.** (**A**) HEK293 cells were co-transfected with 3×HA-NCC, either alone or in combination with a *MAGED2* plasmid, and subjected to knockdown of GNAS (siGNAS) or control (siCtrl) siRNA. At 24–48 h post-transfection, the cells were exposed to hypoxia (1% O_2_) overnight. (**A**) Surface proteins were biotinylated, isolated via streptavidin-agarose precipitation, and analyzed by Western blotting using anti-HA antibodies to detect surface NCC expression. In parallel, total cell lysates were analyzed by SDS-PAGE and immunoblotting to quantify total NCC, *MAGED2*, and Gαs expression. (**C**) HEK293 cells were co-transfected with 3×HA-NCC and either control or *MAGED2* siRNA, treated with 10 µM forskolin (FSK) 48 h post-transfection, and subsequently exposed to hypoxia overnight. (**B**,**D**) Densitometric analysis of the ratio of membrane-bound NCC to total NCC from experiments (**A**,**C**), respectively, with bar graphs representing mean ± SEM. Statistical significance was determined using an unpaired two-sided Student’s *t*-test, ns, not significant, ** *p* ≤ 0.01, *** *p* ≤ 0.001. The results shown are representative of three independent experiments. (**E**) Immunolocalization of NCC in HeLa cells co-transfected with 3×HA-NCC and either control or *MAGED2* siRNA, with or without 10 µM forskolin, under hypoxic conditions. Membrane proteins were biotinylated at 4 °C. Scale bars: 5 μm.

We have recently demonstrated that, under hypoxic conditions, *MAGED2* is essential for the proper localization of Gαs at the plasma membrane, which is crucial for the generation of cAMP and the activation of PKA [8]. In light of this, we questioned whether the reduction in total and membrane-bound NCC in our experiments was a consequence of the impaired function of Gαs. Therefore, we investigated the effects of *MAGED2* overexpression and Gαs depletion on NCC in HEK293 cells subjected to physical hypoxia. As expected, *MAGED2* overexpression promoted total and membrane expression of NCC, whereas the knockdown of Gαs reduced both total and membrane-bound NCC levels, comparable to *MAGED2* knockdown. Interestingly, *MAGED2* overexpression did not fully reverse the effect of Gαs depletion, confirming that Gαs functions downstream of *MAGED2* (Figure 4A,B). To support this notion independently, forskolin, a membrane-bound adenylate cyclase activator acting downstream of Gαs, was employed. HEK293 cells were transfected with either control or *MAGED2* siRNA, treated with FSK, and subjected to physical hypoxia. Immunoblot analysis interestingly demonstrated that FSK fully reversed the effect of *MAGED2* depletion on NCC levels (Figure 4C,D). This observation was corroborated by immunocytochemistry, which demonstrated that NCC relocated to the plasma membrane in *MAGED2*-deficient, hypoxic cells upon FSK treatment (Figure 4E). These results confirm that the effect of *MAGED2* on NCC expression and membrane localization is mediated through the Gαs-cAMP signaling pathway.

### 3.4. MAGED-2 Decreases Exocytotic Insertion of NCC

**Figure 5:**
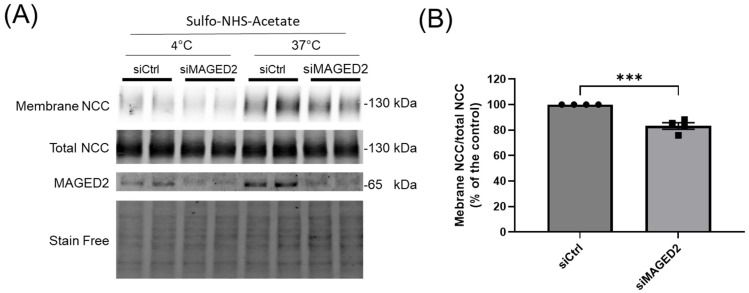
***MAGED2* increases NCC exocytosis.** HEK293 cells co-transfected with 3×HA-NCC and either control or *MAGED2* siRNA. At 24–48 h post-transfection, cells were treated with the hypoxia mimetic CoCl_2_ (300 μM) overnight. Following hypoxia, cells were labeled with sulfo-NHS-acetate at room temperature for 2 h and then incubated at 37 °C for 20 min. After the incubation period, surface proteins were biotinylated using sulfo-NHS -biotin. (**A**) Cells were subsequently lysed, and biotinylated proteins were isolated via streptavidin-conjugated agarose beads and resolved by SDS-PAGE. Surface-labeled NCC and total cell lysates were analyzed by Western blotting using an anti-HA antibody to detect surface and total NCC, and an anti-*MAGED2* antibody to verify knockdown efficiency. (**B**) Densitometric analysis of the ratio of membrane-bound NCC to total NCC representing newly membrane inserted proteins, with statistical significance determined by unpaired two-sided Student’s *t*-test, *** *p* ≤ 0.001. The results shown are representative of four biological replicates.

NCC is mainly located in intracellular vesicles, which are subject to exocytosis and endocytosis [25]. Therefore, the decrease in membrane-bound NCC upon *MAGED2* depletion can be caused by diminished exocytotic insertion, enhanced endocytic retrieval, or a combination of both processes. To determine whether decreased exocytosis contributes to this effect, we measured the rate of exocytotic insertion. At 48 h post-transfection of HEK293 cells with NCC, in the presence or absence of *MAGED2*, extracellular lysine residues reacting with sulfo-NHS-LC-biotin were initially masked by incubating them with membrane-impermeant sulfo-NHS-acetate. Cells were then incubated at 37 °C for 30 min to allow protein trafficking, which was succeeded by the biotinylation of cell surface proteins. In this context, biotinylated NCC represents NCC that was originally present intracellularly (protected from sulfo-NHS-acetate) and had been translocated to the cell surface (Figure S1A). The experimental controls were carried out by omitting the 37 °C step, and any signal obtained indicated that the surface reactive sites were not fully saturated with sulfo-NHS-acetate rather than exocytosis at 4 °C. Therefore, *MAGED2* depletion leads to a reduced rate of exocytic insertion of NCC into the cell surface, as indicated by the lower ratio of membrane-bound NCC to total NCC (Figure 5A,B). These data suggest that the reduction in NCC surface expression by *MAGED2* depletion is due in a part to decreased exocytosis.

### 3.5. MAGED-2 Increases Endocytic Retrieval of NCC

**Figure 6:**
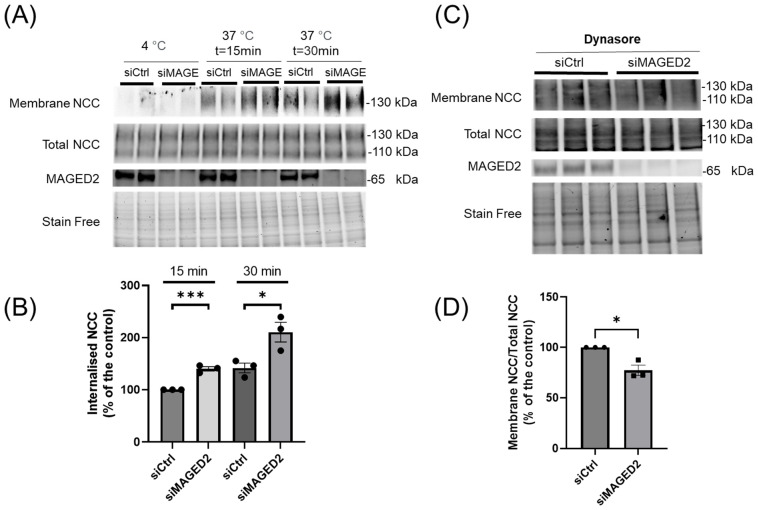
***MAGED2* decreases clathrin-mediated endocytosis of NCC under hypoxic conditions**. (**A**) HEK 293 cells were transfected with the 3×HA-NCC construct in combination with either control siRNA or *MAGED2* siRNA. Cells were exposed to the hypoxia mimetic CoCl_2_ (300 µM) overnight for 24–48 h post-transfection. After the hypoxia treatment, surface proteins were biotinylated using sulfo-NHS-SS-biotin, then incubated at 37 °C for 15 or 30 min to allow endocytosis of labeled proteins. Following the incubation period, surface biotin was removed by treating the cells with L-glutathione, enabling the visualization of the internalized proteins. Cells were then lysed, and biotinylated proteins were purified via streptavidin-agarose beads, resolved by SDS-PAGE, and analyzed by Western blotting. Surface-labeled and internalized NCC were detected using an anti-HA antibody, and *MAGED2* knockdown was verified using an anti-*MAGED2* antibody. (**C**) HEK293 cells were co-transfected with the 3×HA-NCC construct and either control or *MAGED2* siRNA. At 48 h post-transfection, cells were treated with physical hypoxia overnight in the presence of the endocytosis inhibitor Dynasore (50 µM). Surface proteins were biotinylated and isolated from cell extracts using streptavidin-agarose beads. Surface NCC expression was detected by Western blotting with an anti-HA antibody, and total cell lysates were analyzed by SDS-PAGE and immunoblotted with anti-HA and *MAGED2* antibodies. (**B**,**D**) Densitometric analysis of the ratio of membrane-bound NCC to total NCC of experiments (**A**,**C**) respectively, with statistical significance determined by an unpaired two-sided Student’s *t*-test (* *p* ≤ 0.05, *** *p* ≤ 0.001). The results shown are representative of three biological replicates.

We then investigated the effect of *MAGED2* on endocytosis of NCC in HEK293 cells. To quantify endocytic internalization, we employed a glutathione protection assay. At 48 h after transfection of HEK293 cells with NCC, in the presence or absence of *MAGED2*, surface proteins were initially biotinylated with the thiol-cleavable amine-reactive biotinylation reagent sulfo-NHS-SS-biotin. Subsequently, cells were incubated at 37 °C for 15 or 30 min to facilitate endocytosis before the application of glutathione. This approach ensures that biotinylated NCC corresponds to the NCC that was present on the plasma membrane and subsequently internalized, thereby being shielded from glutathione (Figure S1B). To evaluate the effectiveness of glutathione cleavage, control experiments were conducted at 4 °C immediately post-biotinylation (time 0). As shown in Figure 6A,B, *MAGED2* depletion increases the ratio of biotinylated NCC (internalized NCC) to total NCC, suggesting that the decrease in NCC surface expression by *MAGED2* siRNA is partly due to enhanced endocytic internalization. To further elucidate the role of *MAGED2* in regulating NCC endocytosis under hypoxic conditions, we employed dynasore, a dynamin inhibitor, based on previous findings that NCC endocytosis is clathrin-mediated [26]. Treatment with dynasore partially reversed the effect of *MAGED2* depletion on NCC membrane localization (Figure 6C,D).

These findings provide evidence that *MAGED2* modulates NCC surface membrane expression under hypoxia through a synergistic combination of two trafficking processes: increased endocytosis and decreased exocytosis in *MAGED2*-depleted cells.

### 3.6. MAGED2 Depletion Down-Regulates NCC by Enhancing Its Ubiquitination and Lysosomal Degradation

**Figure 7:**
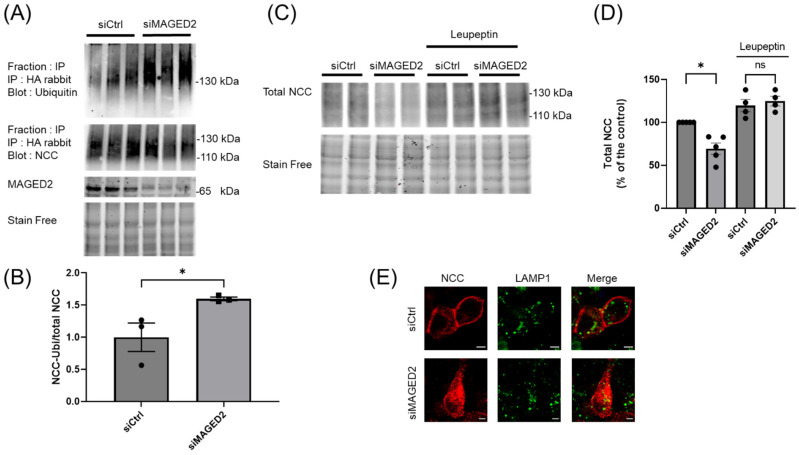
***MAGED2* inhibits NCC ubiquitination and its lysosomal degradation under hypoxic conditions**. (**A**) HEK293 cells co-transfected with 3×HA-NCC and control or *MAGED2* siRNA were immunoprecipitated with anti-HA under denaturing conditions. Ubiquitinated and pulled-down NCC were detected by Western blot using anti-ubiquitin and anti-HA antibodies, respectively. In parallel, total cell lysates were analyzed by SDS-PAGE and immunoblotting to quantify total NCC and *MAGED2* expression. Samples from three independent experiments are loaded in one gel. (**B**) Densitometric analysis of (**A**), shown as the ratio of ubiquitinated NCC and total NCC immunoblot. (**C**) HEK293 cells were co-transfected with 3×HA-NCC and either control or *MAGED2* siRNA. At 24–48 h post-transfection, cells were treated with physical hypoxia overnight in the presence or absence of a lysosomal inhibitor, leupeptin 100 µM. (**D**) Total cell extracts were analyzed by SDS-PAGE and immunoblotting to quantify total NCC and *MAGED2* expression. (**B**,**D**) Densitometric analysis is shown; bar graphs represent mean ± SEM, with statistical significance determined by an unpaired two-sided Student’s *t*-test, ns, not significant, * *p* ≤ 0.05. The results shown (**D**) are representative of four biological replicates. (**E**) Immunolocalization studies of NCC and LAMP1 (a lysosome marker) in the presence or absence of *MAGED2* were conducted under hypoxic conditions. Scale bars represent 5 μm.

The endocytosis of numerous proteins is highly regulated by E3 ubiquitin ligases, with ubiquitin acting as a critical sorting signal for internalization at the plasma membrane. Once internalized, the protein cargo is either directed to recycling endosomes for reappearance on the cell membrane, or it can be routed to the lysosomes for degradation [27,28]. Given that *MAGED2* depletion under hypoxia promotes NCC endocytosis and decreases its abundance, we speculated that NCC undergoes ubiquitination in a *MAGED2*-dependent manner to regulate its internalization and degradation. To investigate this, we expressed 3×HA-tagged NCC in both control and *MAGED2* knockdown conditions, followed by exposure to physical hypoxia. Immunoprecipitation of NCC using an HA-specific antibody, followed by immunoblotting, revealed a marked increase in ubiquitin signal intensity upon *MAGED2* depletion (Figure 7A). Since the ratio of ubiquitin-bound NCC to the immunoprecipitated NCC was higher in the absence of *MAGED2*, our findings strongly suggest that the ubiquitination of NCC is significantly elevated under hypoxic conditions upon *MAGED2* depletion.

To determine if the internalized NCC is targeted for lysosomal degradation in the absence of *MAGED2*, we treated cells with leupeptin, an inhibitor of lysosomal proteases. As shown in Figure 7C,D, the reduction in total NCC levels was reversed upon treatment with leupeptin, indicating that the observed decrease in NCC was due to an increase in lysosomal degradation. This conclusion was further supported by immunofluorescence, which showed colocalization of NCC with the lysosome marker LAMP1 upon *MAGED2* depletion under hypoxia (Figure 7D). In summary, *MAGED2* decreases NCC endocytosis by reducing its ubiquitination, thereby preventing its internalization under hypoxic conditions.

## 4. Discussion

Bartter syndrome type V, the most severe form of BS, is caused by X-linked mutations in *MAGED2*. It is characterized by an impaired expression of the key renal salt transporters *NKCC2* and NCC, leading to severe but transient renal salt wasting and hence excessive fetal urine production (fetal polyuria), resulting in extreme prematurity, and frequently fetal demise [4,5]. Although studies by our group and others demonstrated that PKA-dependent phosphorylation promotes NCC activity [29] and that *MAGED2* secures cAMP generation under hypoxia [8,17], the potential role of *MAGED2* in the regulation of NCC remained unclear. The present study reveals, for the first time, the mechanisms by which *MAGED2* controls NCC trafficking. Under hypoxic conditions and in a cAMP-dependent manner, *MAGED2* depletion decreases NCC exocytosis while increasing its endocytosis. The latter is paralleled by enhanced ubiquitination of NCC, which targets it for lysosomal degradation.

The importance of Gαs signaling in regulating renal salt transporters is well established. For instance, heterozygous Gαs knockout mice exhibit a significant reduction in *NKCC2* abundance, along with decreased cAMP production and impaired urinary concentrating ability [30]. Although the expression of NCC was not assessed in this study, it is noteworthy that NCC, which like *NKCC2* is from the SLC12 family encoding the CCC, shares several upstream regulators with *NKCC2* [31]. Furthermore, the regulation of NCC by vasopressin, and hence cAMP, has been well demonstrated, through its role in modulating NCC phosphorylation and luminal expression in the distal convoluted tubule [29,32,33]. Our findings extend these studies by demonstrating that *MAGED2* regulates NCC trafficking and lysosomal degradation by securing Gαs signaling under hypoxic conditions.

Although overexpression of *MAGED2* significantly increases NCC plasma membrane expression, it only partially compensates for the inhibitory effect of Gαs depletion on NCC membrane expression. These findings clearly show that Gαs is essential for maintaining NCC at the plasma membrane, as its loss can probably only be compensated by cAMP activators such as forskolin. This conclusion is supported by our observation that forskolin fully rescued NCC membrane expression in *MAGED2* depleted cells.

Considering the role of forskolin, a diterpenoid from *Coleus forskohlii* traditionally used in Indian medicine for conditions associated with low cAMP levels as a cAMP activator, may hold therapeutic potential for tBS. Forskolin’s ability to stimulate cAMP has been documented in various conditions, including reduced urine volume in diabetic nephropathy in rats [34], which could potentially support renal function under certain conditions. Importantly, forskolin crosses the placental barrier [35], indicating its potential for therapeutic applications in fetal conditions like tBS where enhancing cAMP could counteract renal salt wasting by promoting NCC and *NKCC2* activity.

The fact that *MAGED2* overexpression enhanced NCC membrane expression in both the presence and absence of Gαs clearly demonstrates that *MAGED2* also regulates NCC through Gαs-independent mechanisms. A potential candidate mediating this Gαs-independent regulation of *MAGED2* is HSP40/DNAJB1, a chaperone protein known to promote the export of NCC from the endoplasmic reticulum [36]. Our previous work identified HSP40/DNAJB1 as one of the two interactors binding to wild type *MAGED2* but not with its mutant form [5]. It is noteworthy that the cAMP/PKA pathway regulates DNAJB1 by increasing its phosphorylation via MK5 (MAP kinase-activated protein kinase 5), which enhances the ATP hydrolysis activity of HSP40/HSP70 complex [37,38]. HSP70 is involved in the regulation of NCC ubiquitination and degradation [26,39]. Our data are compatible with the notion that *MAGED2* may regulate NCC through HSP40 in both cAMP-dependent and -independent manners.

Furthermore, we demonstrated that the reduction in the surface expression of NCC observed with *MAGED2* depletion is due to a combination of decreased exocytic insertion and increased ubiquitin-dependent endocytosis and lysosomal degradation. Our findings align with previous observations in which vasopressin treatment of rats with diabetes insipidus for 30 min, a time course similar to our trafficking assay, resulted in increased NCC luminal expression and phosphorylation [32]. Our results reveal for the first time that cAMP-dependent membrane translocation of NCC can be driven by both increased exocytosis and decreased endocytosis. This dual effect of cAMP on the membrane expression of NCC may be explained by the complex role of the cAMP/PKA pathway in the regulation of NCC modulators. PKA phosphorylates protein phosphatase 1 inhibitor-1 (I-1) and acts as a potent inhibitor of the protein phosphatase 1 (PP1). PP1 can regulate NCC directly or indirectly through its modulators, such as WNK4 or HSP70 [40,41,42]. HSP70 is involved in the regulation of NCC ubiquitination and degradation [26,39]. Besides I-1, PKA phosphorylates Kelch-like 3 (KLHL3) at the S433 site, leading to an increase in the expression levels of WNK4 [43]. WNK4 is involved in the regulations of NCC phosphorylation and membrane expression [44,45,46]. In addition to the phosphorylation of NCC, PKA can regulate NCC ubiquitination by phosphorylating NEDD4-2, leading to the attenuation of its ubiquitin ligase activity [47,48,49,50].

Our findings indicate that *MAGED2* depletion enhances the lysosomal degradation of NCC under hypoxic conditions, which is consistent with our prior research showing that *MAGED2* depletion facilitates stress-induced autophagy in a cAMP-dependent manner [22]. This observation aligns with previous studies demonstrating that internalized NCC can be directed toward lysosomal degradation [51,52,53]. This additional regulatory mechanism emphasizes the complex interplay of signaling pathways that govern NCC activity and underscores the broader implications of cAMP and *MAGED2* in the maintenance of sodium homeostasis in the kidney.

Given the important role of cAMP in promoting activity of not only *NKCC2* and NCC, but also of other transporters [9], the question arises if additional transporters are also affected in tBS. A potential candidate is ENAC given that its loss of function causes hyperkalemia and metabolic acidosis [54], which would explain why tBS patients have normal potassium and bicarbonate values, but not a profound hypokalemic metabolic alkalosis, which would result from dual inhibition of *NKCC2* and NCC alone (as seen, patients subjected to sequential nephron blockade by administering furosemide and thiazide to block *NKCC2* and NCC, respectively [55]).

Renal hypoxia is not only a critical determinant during fetal life, but also plays a significant role postnatally, where it has been extensively studied in human disease and experimental models of acute kidney failure. The proximal tubule, known for its transport activity, is exquisitely sensitive to hypoxia in various types of acute kidney injury. Of interest, *MAGED2* is induced both in acute and chronic ischemia reperfusion in the murine DCT [19], indicating that *MAGED2* might also have a postnatal role to secure NCC functioning under stress conditions.

A possible limitation in this study is that our experiments were context-dependent, utilizing the HEK293 cell line, which may not fully replicate the complexity of human renal physiology. Future studies would benefit from incorporating additional human distal tubule cell lines and exploring human fetal kidney tissues, where feasible. The use of quantitative (qPCR) or semi-quantitative (Western blot) assays on fetal tissues could have provided more comprehensive insights, but were precluded because of limited availability of human fetal tissue. Additionally, investigating other cAMP-sensitive transporters, such as *NKCC2* and ENaC, could further elucidate the severity and broader implications of the syndrome. Addressing these limitations will enhance our understanding of the disease and potentially lead to more effective therapeutic strategies.

In conclusion, our findings unravel the critical role of *MAGED2* in regulating NCC under hypoxic conditions, offering new insights into the pathophysiology of transient Bartter syndrome. The regulation of NCC by *MAGED2* via Gαs signaling, coupled with its impact on protein trafficking, underscores the complexity of this syndrome and highlights potential therapeutic strategies. By targeting the cAMP/PKA pathway involved in the regulation of both NCC and *NKCC2*, it may be possible to mitigate the effects of renal salt wasting in individuals with tBS, improving clinical outcomes for this rare but severe disorder.

## Figures and Tables

**Table 1:** Reagents and tools.

Reagent or Resource	Source	Identifier
**Antibodies**		
Anti-*MAGED2* rabbit raised against this peptide (QVQENQDTRPKVKAK)	Eurogentec (Cologne, Germany)	
Anti-Gαs	Sigma Aldrich (Schnelldorf, Germany)	06-237
Anti-Ubiquitin	Invitrogen (Dreieich, Germany)	13-1600
Anti-HA tag rabbit	Thermo Fisher Scientific (Dreieich, Germany)	71-5500
Anti-HA tag mouse	Thermo Fisher Scientific (Dreieich, Germany)	26183
Goat anti-Mouse IgG (H + L), Alexa Fluor Plus 555	Thermo Fisher Scientific (Dreieich, Germany)	A32727
Streptavidin, Alexa Fluor™ 488 conjugate	Thermo Fisher Scientific (Dreieich, Germany)	S11223
StarBright Blue 520 Goat Anti-Rabbit IgG	Bio-rad (Dreieich, Germany)	12005869
StarBright Blue 700 Goat Anti-Mouse IgG	Bio-rad (Dreieich, Germany)	12004158
**Chemicals, Peptides, and Recombinant Proteins**		
EZ-Link™ Sulfo-NHS-LC-Biotin	Thermo Fisher Scientific (Dreieich, Germany)	21335
Pierce™ Premium Grade Sulfo-NHS-SS-Biotin	Thermo Fisher Scientific (Dreieich, Germany)	PG82077
Pierce™ Sulfo-NHS-Acetate	Thermo Fisher Scientific (Dreieich, Germany)	26777
L-Glutathion reduziert	Roth (Karlsruhe, Germany)	6832.3
Leupeptin hemisulfate	AdipoGen Life Sciences (San Diego, CA, USA)	AG-CP3-7000-M005
Mg-132	Sigma-Aldrich (Schnelldorf, Germany)	474787
Dynamin-Inhibitor I, Dynasore	Sigma-Aldrich (Schnelldorf, Germany)	324410
Forskolin	Sigma-Aldrich (Schnelldorf, Germany)	F6886
CoroNa Green, AM	Thermo Fisher Scientific (Dreieich, Germany)	C36676
Streptavidin-Agarose Resin	Thermo Fisher Scientific (Dreieich, Germany)	20353
Dynabeads Protein G	Thermo Fisher Scientific (Dreieich, Germany)	10004D
Bumetanide	Sigma-Aldrich (Schnelldorf, Germany)	B-3023
Quabain	Sigma-Aldrich (Schnelldorf, Germany)	O-3125
Amiloride	Sigma-Aldrich (Schnelldorf, Germany)	A-7410
Hydrochlorothiazide	Sigma-Aldrich (Schnelldorf, Germany)	H2910
**Critical Commercial Assays**		
Q5^®^ Site-Directed Mutagenesis Kit	New England Biolabs (Frankfurt am Main, Germany)	E0554S
QuikChange Multi Site-Directed Mutagenesis Kit	Agilent Technologies (Santa Clara, CA, USA)	200515
**Experimental Models: Cell Lines**		
HEK293	ATCC	CRL1573
HeLa	Gift from Dr. Vijay Renigunta	
**Oligonucleotides**		
ON-TARGETplus Non-targeting Control Pool	Dharmacon (Lafayette, CO, USA)	D-001810-10-05
UGGUUUACAUGUCGACUAA		
UGGUUUACAUGUUGUGUGA		
UGGUUUACAUGUUUUCUGA		
UGGUUUACAUGUUUUCCUA		
ON-TARGETplus Human *MAGED2* siRNA—SMARTpool	Dharmacon (Lafayette, CO, USA)	L-017284-01-0005
GGACGAAGCUGAUAUCGGA		
GCUAAAGACCAGACGAAGA		
AGGCGAUGGAAGCGGAUUU		
GAAAAGGACAGUAGCUCGA		
ON-TARGETplus Human GNAS siRNA—SMARTpool	Dharmacon (Lafayette, CO, USA)	L-010825-00-0005
GCAAGUGGAUCCAGUGCUU		
GCAUGCACCUUCGUCAGUA		
AUGAGGAUCCUGCAUGUUA		
CAACCAAAGUGCAGGACAU		
NCC 3×HA primer	Sigma-Aldrich (Schnelldorf, Germany)	
CCCGGACTATGCAGGATCCTATCCATATGACGTTCCAGATTACGCTATGGCAGAACTGCCCACA		
ACGTCATAGGGATAGCCAGCGTAATCTGGAACATCGTATGGGTACATGGATCCGAATTCGCCCTATAG		
**Recombinant DNA**		
pCMV5-HA-1 SLC12A3 (hNCC)	University of Dundee	DU4461
pTargeT	Promega (Walldorf, Germany)	A1410
**Software and Algorithms**		
ImageJ 1.51j8	[20]	https://imagej.nih.gov/ij/ (accessed on 25 Fabruary 2022)
GraphPad Prism 9	GraphPad	
EndNote 21	Clarivate Analytics	
ZEN Microscopy Software 3.3	ZEISS Microscopy	
BioRender	BioRender	www.biorender.com (accessed on 25 Fabruary 2024)

## Data Availability

The original contributions presented in this study are included in the article/Supplementary Material. Further inquiries can be directed to the corresponding authors.

## Supplementary Materials

The following supporting information can be downloaded at: www.mdpi.com/article/10.3390/cells14030175/s1, Figure S1: Exocytosis (A) and endocytosis (B) experimental setup.
