# Supplementary figures and images for: MAGED2 Enhances Expression and Function of NCC at the Cell Surface via cAMP Signaling Under Hypoxia
Source: Cells. 2025 Jan 23;14(3):175. doi: 10.3390/cells14030175 (PMC11818053; doi:10.3390/cells14030175)

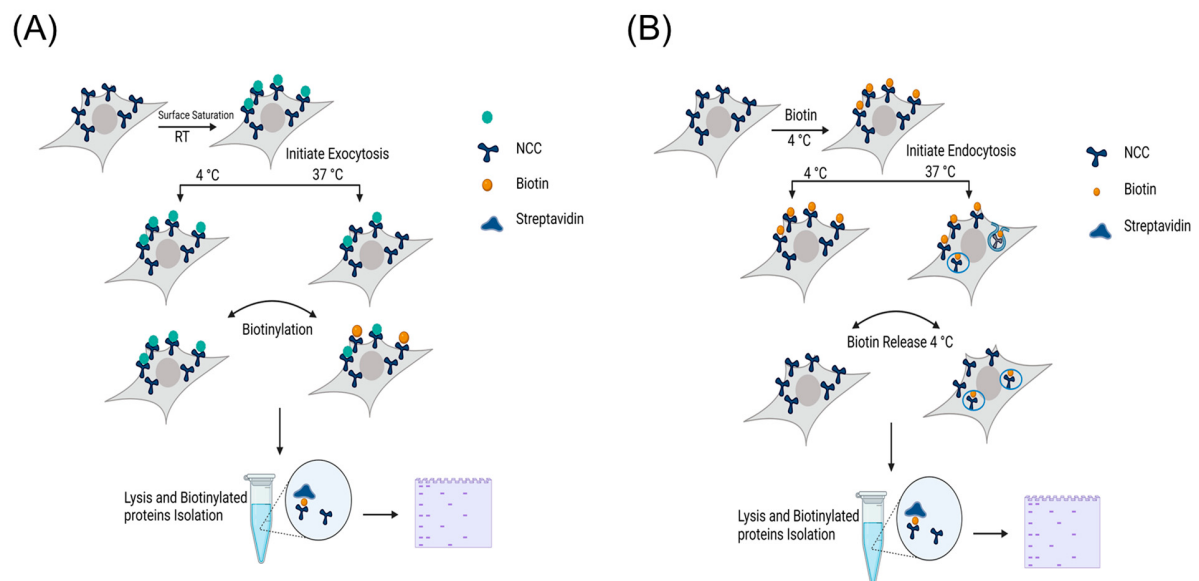

Figure S1. **Exocytosis (A) and endocytosis (B) experimental setup.** (created with biorender.com)

Supplement: Supplementary file 1 [file cells-14-00175-s001.zip › cells-3378507-supplementary.pdf]
